# Paeoniflorin, the Main Active Ingredient of Shuyu Capsule, Inhibits Ca_v_1.2 and Regulates Calmodulin/Calmodulin-Dependent Protein Kinase II Signalling

**DOI:** 10.1155/2017/8459287

**Published:** 2017-12-06

**Authors:** Chunhong Song, Jieqiong Wang, Dongmei Gao, Yanhong Yu, Fang Li, Sheng Wei, Peng Sun, Meiyan Wang, Mingqi Qiao

**Affiliations:** ^1^Experiment Center, Shandong University of Traditional Chinese Medicine, Jinan 250355, China; ^2^College of Pharmacy, Shandong University of Traditional Chinese Medicine, Jinan 250355, China; ^3^School of Preclinical Medicine, Shandong University of Traditional Chinese Medicine, Jinan 250355, China; ^4^Fengtai Maternal and Children's Health Hospital of Beijing, Beijing 100069, China

## Abstract

The aim of this study was to explore the mechanism underlying the antidepression activity of paeoniflorin, the main active ingredient of paeony extract and Shuyu capsules, and determine its effect on the calmodulin/calmodulin-dependent protein kinase II (CaM/CaMKII) signalling pathway and on the possible target, the voltage-gated calcium channel (Ca_v_). Rats at the nonacceptance stage were selected for premenstrual syndrome (PMS) depression modelling. Behavioural assays were used for model testing. Rats were given Shuyu capsules, paeony extract, and bupleurum. Western blot analysis was used to assess the expression levels of calcium voltage-gated channel subunit alpha 1 C (CACNA1C), brain-derived neurotrophic factor, and CaM/CaMKII signalling pathway proteins. Intracellular Ca^2+^ concentration in CHO cell line was measured using Fluo-4-AM and whole-cell patch clamps. The PMS depression model was successfully established and demonstrated that Shuyu can mitigate depressive behaviour in a rat PMS model. Paeony extract did not affect CACNA1C protein expression in rat hippocampi but did affect Ca_v_1.2-mediated CaM/CaMKII signalling pathways. Paeoniflorin significantly inhibited KCl-induced increases in intracellular Ca^2+^ concentration and Ca_v_1.2 current density. Further, it may function via the CaM/CaMKII pathway and its downstream signalling molecules by regulating Ca_v_1.2, thus playing an important role in the treatment and alleviation of affective disorders.

## 1. Introduction

Premenstrual syndrome (PMS), commonly encountered in clinical gynaecology, refers to a series of mental and physical symptoms, including anxiety, breast pain, and headaches, which occur during the premenstrual period (luteal phase) in 20~50% of reproductive-aged women [1]. Symptoms lessen or disappear after menstruation but recur with each menstrual cycle [2, 3]. Epidemiological surveys conducted worldwide suggest that reproductive-aged women experience one or more PMS-related symptoms [4] that significantly influence their health and quality of life. PMS depression is characterized by symptoms including negative affect (depression, anger, aggression, crying spells, mood swings, tension, irritability, and anxiety), water retention, and pain [5, 6], and the incidences of these symptoms have recently increased, attracting increased attention from medical fields.

Shuyu, a commercially available herbal prescription of traditional Chinese medicine, comprises four herbal ingredients: Radix Bupleuri (*Bupleurum chinense* DC.), Radix Paeoniae Alba (*Paeonia lactiflora* Pall.), Rhizoma Cyperi (*Cyperus rotundus* Linn.), and Radix Glycyrrhizae (*Glycyrrhiza uralensis* Fisch.). Studies have shown that Shuyu can mitigate PMS depression symptoms and that its action mechanism is concentrated in specific cerebral areas [7, 8]. Following intragastric administration of Shuyu or paeony extract, paeoniflorin, one of the constituents, is absorbed into the blood [9]. Paeoniflorin, the main component of Shuyu and paeony extract, has many biological effects, including enhancement of cognitive ability, improvements in learning disabilities, and nerve protection [10–12], suggesting that it may be the main active substance of Shuyu capsule that has psychological and, therefore, antidepressant, effects. However, the mechanism by which paeoniflorin exerts its antidepressant effect is still poorly understood.

Calcium dysregulation could be related to PMS and researches revealed a significantly decreased Ca^2+^ concentration in the blood corpuscles and serum of PMS patients [1, 13]. Voltage-gated calcium channels regulate intracellular Ca^2+^ concentration.* CACNA1C* encodes the *α*1C subunit of the L-type voltage-gated calcium channel (LTCC), known as Ca_v_1.2, which is the main subunit of LTCC. Ca_v_1.2 is a transmembrane channel protein and contains the binding sites for dihydropyridine drugs. In recent years, the relationship between* CACNA1C* and affective disorder has been widely studied. Genomewide association studies show that* CACNA1C* polymorphisms are closely related to depression, schizophrenia, and bipolar disorder and are causes of various affective disorders, making them a potential new target for treating mental and emotional disorders [14]. High levels of intracellular Ca^2+^, regulated by Ca_v_, increase the levels of Ca-bound calmodulin (CaM), which in turn activates calmodulin-dependent protein kinase II (CaMKII), which is involved in regulating the synthesis and release of proteins and neurotransmitters including brain-derived neurotrophic factor (BDNF), 5-hydroxytryptamine, dopamine, and glutamate, all of which are related to the incidence of PMS. In this study, paeoniflorin was applied to CHO cells stably expressing Ca_v_* in vitro*, and a PMS depression rat model was used to explore the mechanism by which paeoniflorin exhibits an antidepressant effect. In particular, we studied the expression of key proteins in the Ca_v_ downstream signalling pathway under paeoniflorin treatment.

## 2. Materials and Methods

### 2.1. Laboratory Animals and Ethics Statement

Healthy female SPF Wistar rats weighing 140–160 g were selected and given free access to water and food. The feeding room temperature was 24 ± 1°C and the relative humidity was 50 ± 10%. The rats were provided by the Laboratory Animal Center of Shandong Traditional Chinese Medicine University, License Number SCXK (LU) 2011-0003. Fifty rats were randomized into five groups (*n* = 10) including a control group, model group, Shuyu treatment group, paeony extract treatment group, and bupleurum extract treatment group. Rats in the control group were not stimulated while those in the model group were stimulated with leg binding (details below). In the Shuyu capsule, paeony extract, and bupleurum extract treatment groups, medicine was chronically administered to rats during the modelling period. Rats in each experimental group were weighed at consistent intervals during the study.

Hippocampal primary neurons were separated from newborn (24-h-old) Wistar mother rats (provided by Jinan Pengyue Experimental Animal Breeding Co., Ltd., License Number SCXK (Lu) 2014-0007). Laboratory animals were provided with care according to “*The Care and Use of Laboratory Animals*” by the Laboratory Animal Center of Shandong University of Traditional Chinese Medicine.

### 2.2. Antibodies, Drugs, and Chemicals

Primary antibodies against CaM (SAB4503194, Sigma-Aldrich, St. Louis, MO, USA), CaMKII (4436, Cell Signaling Technology, Danvers, MA, USA), p-CaMKII (Thr286) (3361, Cell Signaling Technology), CACNA1C (ab58552, Abcam, Cambridge, MA, USA), BDNF (AV41970, Sigma-Aldrich), and tubulin (M2005, Abmart, Arlington, MA, USA) were used at dilution ratios recommended in the instruction manual. The secondary antibody, peroxidase-conjugated goat anti-rabbit antibody IgG (Santa Cruz Biotechnology, Dallas, TX, USA), was diluted at a dilution ratio of 1 : 2000.

The Shuyu capsules (clinical approval number 2011L06107), paeony extract (clinical approval number 20110527), and bupleurum extract (clinical approval number 20110526) used in the experiments were intragastrically administered to animals at doses of 0.41 g/kg/d, 0.32 g/kg/d, and 36 mg/kg/d, respectively, for 5 d. During modelling, all groups were given the drug once a day at 9:00 am at a dosage equivalent to 8 times the dosage administered to humans.

Paeoniflorin (Z110736) and nifedipine (N7634-1G) standards were purchased from Shanghai Yuanye Biotech (Shanghai, China) and Sigma-Aldrich, respectively. All other chemicals were obtained from Sinopharm Chemical Reagent Co., Ltd. (Shanghai, China).

### 2.3. Determination of the Oestrous Cycle

All rats were weighed and marked with picric acid. The oestrous cycle was determined by microscopic examination of vaginal smears [15, 16]. In a prooestrus vaginal smear, epithelial cell nuclei and keratinocytes were observed, while in an oestrus vaginal smear, anucleate keratinocytes and epithelial cells were observed. In a postoestrus vaginal smear, leukocytes, keratinocytes, and epithelial cell nuclei were observed, and in an anoestrus vaginal smear, many leukocytes and few epithelial cells and myxocytes were observed.

### 2.4. Generation of a PMS Depression Rat Model

Rats tend to be active at the acceptance stage (prooestrus and oestrus), and their oestrous behaviours abate or disappear at the nonacceptance stage (postoestrus and anoestrus). Rats with regular oestrus behaviours at the nonacceptance stage, with similar open-field tests and sucrose preference test scores, were selected for the trial.

The model was generated according to a previously published protocol, with some modifications [8]. The rats' legs were bound crosswise; the front and hind leg on the opposite side were bound with a sterile gauze (width: 2 cm) to prevent free movement but in a way that they could move slightly and obtain food. The same amount of sterile drinking water was provided to the experimental and control groups. Modelling lasted for 5 d.

### 2.5. Behavioural Assays

Locomotor activity in rats was measured using an open-field test [17, 18] with an XR-Xvideo (including the XR-Xvideo animal behaviour analytical system). Under dim red light, experimenters placed the distal third of the rat tails at the centre of an open-field test box (size: 50 × 50 × 40 cm) with black walls and a black floor. The system recorded behavioural changes and general movement over 5 min. The model test was conducted after 5 d of modelling and simultaneous drug administration.

Furthermore, the sucrose preference experiment [19] was used to measure the reward response in the rats [20]. Depressive animals show a general decline in sucrose preference, reflecting the symptom of anhedonia in humans. In the experiment, two water bottles were provided for free selection over 24 h. The two bottles contained a 1% aqueous sucrose solution, and pure water, respectively, and were located on opposite sides of the cage. Before the experiment, the rats had free access to water and food. Consumption of each water type was measured based on bottle weight. Sucrose preference was expressed as the percentage of consumed sucrose water to the total liquid consumed. Sucrose preference rate was calculated using the following formula: sucrose water consumption (g)/[sucrose water consumed (g) + water consumption (g)] × 100%.

### 2.6. Western Blotting

Western blot analyses were performed according to a previously published protocol [21]. Band intensities were determined by densitometry analysis using Image J software (National Institutes of Health, Bethesda, MD, USA), and the results were expressed as a ratio of target protein to tubulin or as the ratio of phosphorylated protein to total protein amount.

### 2.7. Primary Culture of Hippocampal Neurons

Twenty-four-hour-old Wistar rats were decapitated and the heads were placed into iced phosphate-buffered saline (PBS). The hippocampus was removed and placed into 10 mL of PBS containing ice. Trypsin (0.25%) was added, and the specimens were incubated at 37°C for 20 min with the tubes shaken every 5 min. Trypsinized cell samples were collected and washed in Dulbecco modified Eagle's minimal essential medium (DMEM) three times. After 4 h, the medium was replaced with NBG medium (neurobasal medium : B27 : L-glutamine = 100 : 2 : 1, Gibco/Life Technologies, Carlsbad, CA, USA) for incubation. The medium was replaced after 24 h and then replaced every 3.5 d.

### 2.8. Changes in Intracellular Ca^2+^ Concentration

Hippocampal neurons* in vitro* were divided into four groups after being cultured for 7 d: blank control group (0 *μ*M), high-dose paeoniflorin group (200 *μ*M), middle-dose paeoniflorin group (100 *μ*M), and low-dose paeoniflorin group (50 *μ*M). The medium from each group was aspirated and the cells were rinsed three times with Hawk's Balance Salt Solution (HBSS, Biotop, Suzhou, China). Fluo-4-AM dye (1 mL, 5 *μ*M; Molecular Probes Life Technologies, Thermo Fisher Scientific Inc., Waltham, MA, USA) was added followed by incubation at 37°C for 45 min. The extracellular residual dyes were washed away with HBSS, and 1 mL of HBSS was added followed by incubation for 15 min. The fluorescence intensity of intracellular Ca^2+^ was measured by laser scanning confocal microscopy (LSM510, ZEISS, Germany) with an exposure time of 100 ms and excitation and emission wavelength at 488 nm and 530 nm, respectively. The images were collected every 3 s for a total duration of 240 s. At the 24th second, KCl was added to the cells of each group making the final concentration 100 mM. At the 80th second, paeoniflorin was added to the cells of each group to detect changes in the fluorescence intensity of intracellular Ca^2+^.

### 2.9. Whole-Cell Patch Clamp

Chinese hamster ovary (CHO) cells stably expressing LTCCs were purchased from Ice Ion Channel Explorer (ICE-CHO- Ca_v_1.2, Beijing, China) and cultured in a standard manner [22]. The voltage clamp mode of the Axon MultiClamp 700B (Molecular Devices, Sunnyvale, CA, USA) was used to measure intracellular voltages. The cell membrane voltage clamp was set at −60 mV when the tight-seal whole cell was formed. The clamping voltage was depolarized from −60 mV to +10 mV for 0.3 s, and the data were collected repeatedly every 20 s with EPC-10 amplifier (HEKA) and stored in the PatchMaster (HEKA) software to observe the influence of paeoniflorin on the electric current peak of L-type calcium channel.

Capillary glass tubes (BF 150-86-10, Sutter Instruments) were pulled into recording electrodes using a pipette puller (P97, Sutter, Sacramento, CA, USA), and the pipette manipulator (MP285, Sutter Instruments) was manipulated under an inverted microscope (IX71, Olympus, Tokyo, Japan) to get recording electrodes in contact with the cells to impose negative pressure suction, thus forming GΩ sealing-in. Fast capacitance compensation was then conducted, and negative pressure was applied to lyse the cell membrane, thus forming a whole-cell recording mode. Slow capacitance compensation was then conducted, and membrane capacitance and series resistance were recorded. However, electric leakage compensation was not provided.

After current stabilization of L-type calcium channel recorded by whole cells, each group (the blank control group, high-dose paeoniflorin group, middle-dose paeoniflorin group, low-dose paeoniflorin group, and nifedipine group) was given the trial drugs, exerting an effect for approximately 5 min. The cell-cover glass was placed in a recording bath under an inverted microscope, and the tested drugs and drug-free external fluid flowed through the recording chamber in the order of gravity perfusion to act on the cells. Fluid exchange was conducted in the recording using a vacuum pump exchange. The current detected in the compound-free extracellular fluid of every cell was taken as its own control group, and multiple cells were independently detected. All electrophysiological experiments were conducted at room temperature (21 ± 1°C).

The current treated by paeoniflorin at certain concentrations was first standardized [peak current of the concentration (pA)/peak current of blank control (pA)], and then the inhibition ratio was calculated [1 − (peak current of the concentration (pA)/peak current of blank control (pA))].

### 2.10. Statistical Analysis

One-way analysis of variance (ANOVA) was used for the weight test, open-field test, and the sucrose preference test. GraphPad Prism 6 was used for the calculations. Photo analyses of hippocampal neurons were performed using the default Zeiss LSM Image Browser software. PClamp 10.0 was used for the whole-cell patch clamp recording of the L-type calcium channel current. All data are shown as the mean ± SD, with the significance level set at *P* < 0.05.

## 3. Results

### 3.1. Shuyu Capsules Effectively Mitigate Depressive Behaviour in a Rat PMS Model

**Figure 1:**
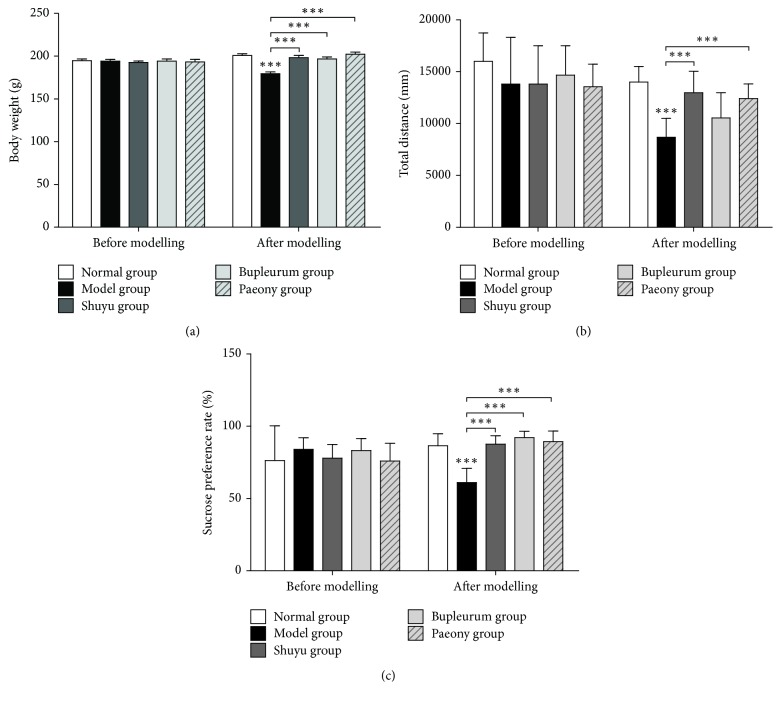
Behavioural assays. (a) Body weight test. (b) Open-field test. (c) Sucrose preference test. For all assays, testing was performed both before and after modelling. Moreover, the following groups were analysed: the control/normal group, model group, Shuyu capsule group, bupleurum group, and paeony group. The statistical analysis for the behaviour assays was performed by one-way ANOVA (*n* = 10, ^*∗∗∗*^*P* < 0.001).

Rat body weights in each group before and after modelling are shown in Figure 1(a). Before modelling, no significant differences in body weight were seen, while after modelling, rats in the model group showed a significant decrease in body weight compared to that shown by the normal group rats (*P* < 0.001), but there were no significant differences between the rats in the treatment groups. Compared to the model group rats, rats in the Shuyu, bupleurum, and paeony groups showed significantly increased body weight (*P* < 0.001).

Results of the open-field test are shown in Figure 1(b). Before modelling, the open-field test scores in each group were not significantly different, while after modelling the open-field test scores of rats in the model group decreased significantly (*P* < 0.001) compared to those of the normal group. The open-field test scores of rats in the Shuyu and paeony groups increased significantly (*P* < 0.001), and those of rats in the bupleurum group showed a tendency to increase, but it was not statistically significance.

Results of the sucrose preference test are shown in Figure 1(c). Before modelling, there was no significant different in the consumption of sucrose water between the groups, while after modelling rats in the model group showed a significant decrease in sucrose preference compared to that shown by the normal group rats (*P* < 0.001). The results for the three treatment groups were not significantly different from those for the normal group. Compared with the model group, each treatment group showed a significant increase in sucrose preference (*P* < 0.001).

### 3.2. Paeony Extract Does Not Affect CACNA1C Protein Expression in the Rat Hippocampus but Can Affect Ca_v_1.2-Mediated CaM/CaMKII Signalling

**Figure 2:**
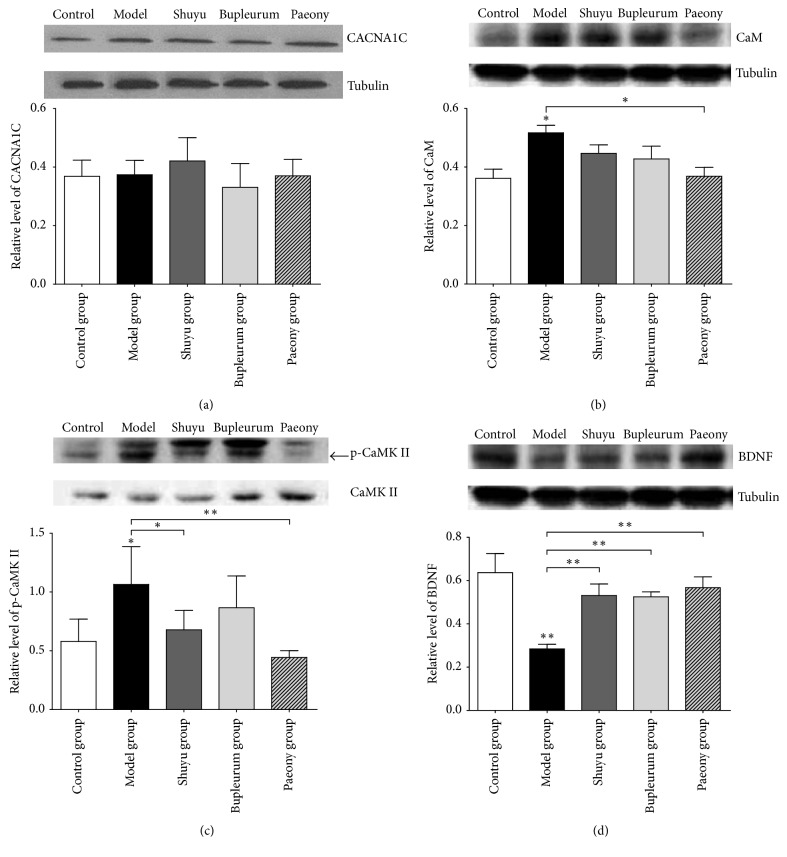
Western blot and analysis of hippocampus tissue samples from the control/normal group, model group, Shuyu capsule group, bupleurum group, and paeony group. (a) CACNA1C protein level analysis (*n* = 6). (b) Analysis of calmodulin (CaM) protein level (*n* = 6, ^*∗*^*P* < 0.05). (c) Analysis of phosphorylated calmodulin-dependent protein kinase II (p-CaMKII) level (*n* = 6, ^*∗*^*P* < 0.05 and ^*∗∗*^*P* < 0.01). (d) Analysis of brain-derived neurotrophic factor (BDNF) protein expression (*n* = 3, ^*∗∗*^*P* < 0.01).

*CACNA1C* expression in the hippocampus is shown in Figure 2(a). Compared with the normal group, the model and treatment groups showed no significant differences in hippocampal* CACNA1C* expression.

Compared with the normal group, the model group showed increased CaM expression in the hippocampus (*P* < 0.05), and there were no significant differences in the hippocampi of treatment group rats. CaM expression in rats of the paeony group was significantly lower than that in rats of the model group (*P* < 0.05, Figure 2(b)).

Expression of phosphorylated CaMKII (p-CaMKII) in model group rats was significantly higher than that in normal group rats (*P* < 0.05). p-CaMKII expression in Shuyu and paeony group rats was significantly lower than that in model group rats (*P* < 0.05, *P* < 0.01, Figure 2(c)).

BDNF expression in the hippocampi of model group rats was significantly lower than that in normal group rats (*P* < 0.01), and there were no significant differences between the treatment groups. BDNF expression was significantly higher in the Shuyu group, bupleurum group, and paeony group rats than in model group rats (*P* < 0.01, Figure 2(d)).

### 3.3. Paeoniflorin Significantly Inhibits KCL-Induced Increases in Intracellular Ca^2+^ Concentration

**Figure 3:**
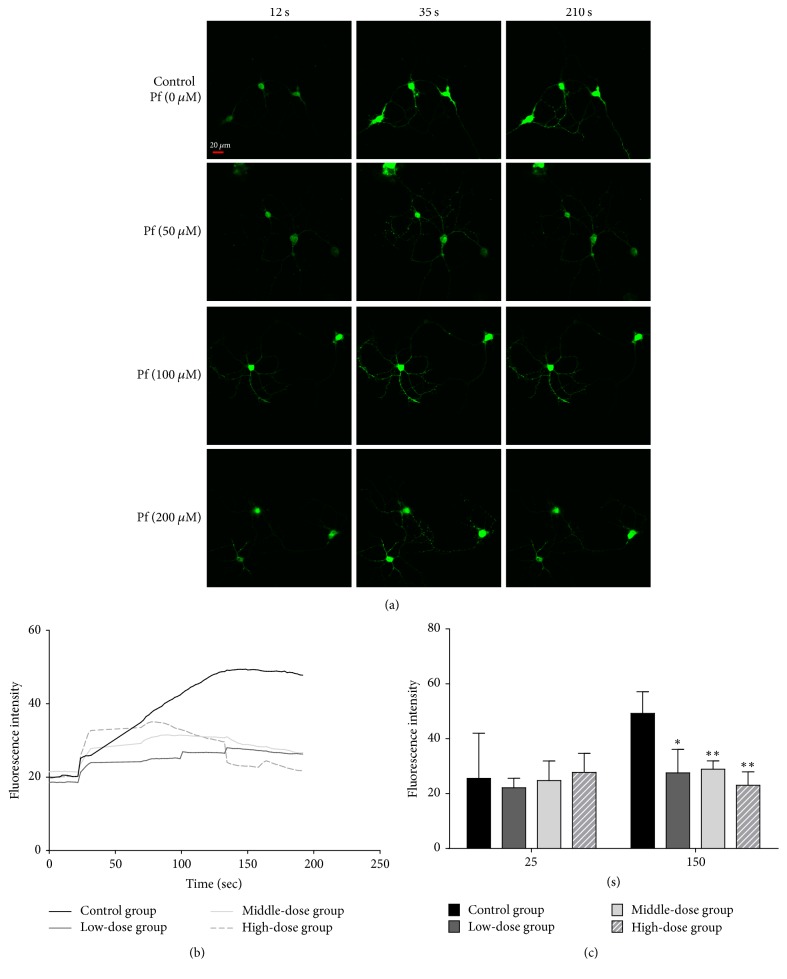
Intracellular calcium ion concentrations of primary culture of hippocampal neurons from groups including blank control, 50 *μ*M paeoniflorin treatment, 100 *μ*M paeoniflorin treatment, and 200 *μ*M paeoniflorin treatment. (a) Morphology and fluorescence intensity of hippocampal neurons in each group before and after KCl and paeoniflorin treatment. Pf: paeoniflorin treatment. (b) Fluorescence intensity of each group over time (0–191 s). (c) Statistical comparison of fluorescence intensity at 25 s and 150 s in each group (*n* = 5, ^*∗*^*P* < 0.05 and ^*∗∗*^*P* < 0.01). Scale bar: 20 *μ*m.

Fluo-4-AM is an acetyl methyl ester derivative of Fluo-4 and is readily accessible from the medium. Upon entering a cell, AM is hydrolysed by intracellular esterase, and then the resultant Fluo-4 binds with Ca^2+^ and emits fluorescence. Fluo-4-AM is therefore used to probe Ca^2+^ levels. Before adding KCl to primary hippocampal neurons, the basal fluorescence intensity of each group was the same, but after KCl was added, intracellular Ca^2+^ increased due to the depolarization of the cell membrane induced by extracellular K^+^, and thus the fluorescence intensity of each group increased sharply (Figures 3(a) and 3(b)). Paeoniflorin intervention inhibited intracellular Ca^2+^ overloading induced by K^+^. Compared with the blank control group, the paeoniflorin treatment group showed a remarkable concentration-dependent decrease in fluorescence intensity (Figures 3(a) and 3(c)), as shown in Figure 3(b).

### 3.4. Paeoniflorin Inhibits Ca_v_1.2 Calcium Channels

**Figure 4:**
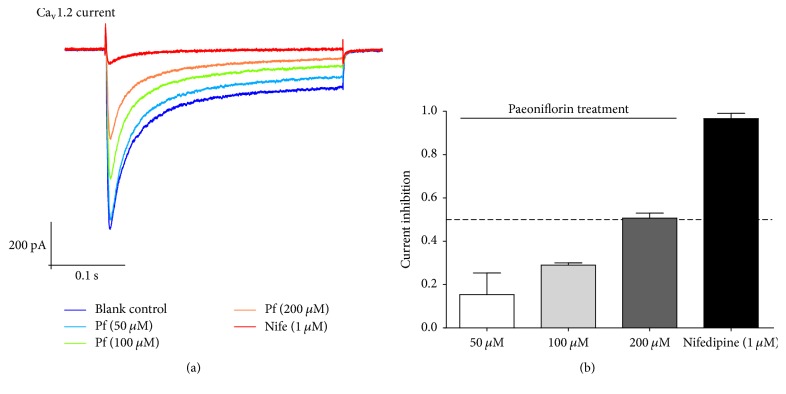
Whole-cell patch clamp analysis of CHO cells with LTCC constitutive expression. (a) Ca_v_1.2 current density was recorded and analysed for the following groups: blank control, positive control (1 *μ*M nifedipine treatment), 50 *μ*M paeoniflorin treatment, 100 *μ*M paeoniflorin treatment, and 200 *μ*M paeoniflorin treatment. (b) Inhibition ratios for 1 *μ*M nifedipine treatment, 50 *μ*M paeoniflorin treatment, 100 *μ*M paeoniflorin treatment, and 200 *μ*M paeoniflorin treatment.

Compared to that of the normal group, the Ca_v_1.2 current density of CHO cells treated with paeoniflorin decreased (*P* < 0.05) in a concentration-dependent manner; the higher the concentration of paeoniflorin, the greater the inhibition of Ca_v_1.2 current density (Figure 4(a)). Nifedipine was used as a positive control and showed nearly 100% inhibition (96.7 ± 4.2%) of Ca_v_1.2 current density. High-dose (200 *μ*M), middle-dose (100 *μ*M), and low-dose paeoniflorin (50 *μ*M) produced 50.7 ± 4.2%, 29.0 ± 1.7%, and 15.3 ± 10.0% inhibition of Ca_v_1.2 current density, respectively (Figure 4(b)).

## 4. Discussion

Postmenstrual symptoms are typical emotional disorders. We demonstrated that postmenstrual symptoms occur in the premenstrual phase and cease postmenstruum in a rat model. Specifically, rats showed symptoms at the nonacceptance period (premenstruum) in the oestrous cycle that disappeared or resolved at the acceptance period (postmenstruum). Other groups verified PMS or premenstrual dysphoric disorder models using similar strategies [23–25]. We selected healthy female Wistar rats through vaginal smear screening, established a PMS depression rat model by means of constraint [26], and studied the animals in a body weight gain test, open-field test, and sucrose preference test. Reduced weight gain in model rats suggested that emotional stress and chronic unpredictable mild stress have the same inhibitory effects on weight gain [27]. The open-field test score measured exploratory behaviour and excitability. The sucrose preference of rodents reflected the reward response; depressive animals commonly show a reduced sucrose preference [28]. The significance of the rat's path, sucrose preference level changes (Figure 1), and some core depression symptoms, during the PMS period were well modelled. Macroscopic behavioural experiments also showed that the administration of Shuyu capsule or paeony extract could relieve the symptoms caused by modelling (Figure 1), indicating that both the Shuyu capsule and the paeony extract contain important molecules that can effectively mitigate depressive behaviour in a rat PMS model.

Many works reported that the hippocampus was related to emotional disorders. Imaging data revealed that patients with depression had smaller hippocampus than that of healthy subjects. Further, the degree of reduction in hippocampus volume was positively correlated with the time for which depression lasted [29]. Another research revealed the relationship between hippocampal morphometry in depressed patients and that in control subjects [30]. As a result, in this study, we measured CaM, CaMKII, p-CaMKII, and BDNF protein levels in the hippocampus of rats in the model and treatment groups. The results showed that neither modelling nor treatment led to a difference in CACNA1C expression levels but that they caused an increase in key protein CaMKII phosphorylation in the Ca_v_1.2-induced CaM/CaMKII signalling pathway and a decrease in BDNF protein expression in model group rats. When Shuyu capsule or paeony extract was administered simultaneously with modelling, the above proteins in the hippocampus of rats were restored to their original levels. Previous studies have demonstrated that exogenous BDNF has an antidepressant activity [31, 32], and this study demonstrates that antidepressant drugs (and their ingredients) can relieve the modelling-induced reduction in BDNF protein in the hippocampus (Figure 2(d)). In addition, p-CaMKII then activates cAMP response element-binding protein (CREB) [33–35], which can regulate the expression of BDNF [36]. In our study, modelling increased CaM and p-CaMKII expression, and administration of Shuyu capsule or paeony extract restored the expression of CaM and p-CaMKII to normal levels. These results indicate that modelling caused an abnormality in the CaM/CaMKII signalling pathway and further affected downstream nerve regulation, while Shuyu capsule (or its ingredients) achieved an antidepression effect by alleviating the pathway abnormality.

Specifically, western blot results suggested that bupleurum extract did not exert effects on Ca_v_1.2-induced CaM/CaMKII signalling pathway (Figure 2). BDNF level also increased in the model group rats treated with bupleurum extract, which exhibited the same effects as paeony extract or Shuyu capsules did. Hence, we further studied the effect of paeoniflorin, the main active molecule in paeony, on Ca^2+^ channel. The Ca^2+^ channel is a transmembrane structure that controls the entry of Ca^2+^ into cells [14]. CACNA1C encodes the *α*1C subunit of the LTCC (Ca_v_1.2 subtype), which is the main subunit of LTCC and is also the target of many antidepressant drugs. CACNA1C acts as a transmembrane channel protein; therefore, extracellular drugs can utilize it to conduct functional intervention of intracellular regulatory factors, thus influencing their physiological functions. Neither modelling nor administration resulted in a difference in CACNA1C expression level, but they did affect the downstream pathway (Figure 2), which suggests that the active molecules in Shuyu capsules may directly act on CACNA1C, although this inference has not been confirmed. Therefore, we further examined the behaviour and function of primary cultured calcium channels under drug intervention. Our previous study showed that paeoniflorin is the main substance of Shuyu capsule [37] to cause an antidepressive effect and that it passes easily through the blood-brain barrier [9]; therefore, we incubated primary hippocampal cells directly with paeoniflorin and observed changes in intracellular Ca^2+^ levels. Our results provide compelling evidence that paeoniflorin can inhibit the overloading of intracellular Ca^2+^ (Figure 3). Further, patch clamp experiments more accurately confirmed this through the constitutive expression of LTCC in CHO cell lines, where paeoniflorin has been shown to inhibit Ca_v_1.2 current density in a concentration-dependent manner (Figure 4). Although other studies as well as ours suggest that paeoniflorin acts on Ca_v_1.2 and influences the CaM/CaMKII pathway, there is not yet enough evidence to confirm this. The influence of paeoniflorin on Ca_v_1.2, the CaM/CaMKII signalling pathway, and the downstream molecules may be concomitant rather than causal; therefore, more studies on this topic are needed.

## 5. Conclusion

This study further evaluates the mechanism of Shuyu capsule with regard to its therapeutic effect against PMS. Paeoniflorin in Shuyu capsules may influence the CaM/CaMKII signalling pathway and its downstream signalling molecules by regulating Ca_v_1.2 current density, thus playing an important role in the treatment and alleviation of affective disorders.
